# *Xiao-Xu-Ming* Decoction Protects against Blood-Brain Barrier Disruption and Neurological Injury Induced by Cerebral Ischemia and Reperfusion in Rats

**DOI:** 10.1155/2013/629782

**Published:** 2013-04-24

**Authors:** Rui Lan, Jun Xiang, Guo-Hua Wang, Wen-Wei Li, Wen Zhang, Li-Li Xu, Ding-Fang Cai

**Affiliations:** ^1^Department of Integrative Medicine, Zhongshan Hospital, and Laboratory of Neurology, Institute of Integrative Medicine, Fudan University, Shanghai 200032, China; ^2^Longhua Hospital, Shanghai University of Traditional Chinese Medicine, Shanghai 200032, China

## Abstract

*Xiao-Xu-Ming* decoction (XXMD) is an effective prescription in the treatment of ischemic stroke, but the mechanisms involved are not well known. In the present study, 120 male Sprague-Dawley rats were randomly divided into 5 groups: sham control (sham), ischemia and reperfusion (IR), and IR plus 15, 30, and 60 g/kg/day XXMD. The stroke model was induced by 90 min of middle cerebral artery occlusion followed by reperfusion. The brain lesion areas were evaluated by 2,3,5-triphenyltetrazolium chloride staining, and neurological deficits were observed at different time points after reperfusion. Blood-brain barrier (BBB) disruption was evaluated by assessing brain water content and Evans blue content. Pathological changes in BBB ultrastructure were observed with transmission electron microscopy. MMP-9, -2, and VEGF expression levels were quantitatively determined by western blotting and immunohistochemistry. We found that XXMD (60 g/kg/day) treatment reduced cerebral infarct area, improved behavioral function, and attenuated ultrastructure damage and permeability of BBB following ischemia and reperfusion. Moreover, XXMD downregulated the expression levels of MMP-9, -2, and VEGF. These findings indicate that XXMD alleviates BBB disruption and cerebral ischemic injury, which may be achieved by inhibiting the expression of MMP-9, -2, and VEGF.

## 1. Introduction

Stroke is a major cause of death and disability worldwide. Numerous researchers have suggested, based on new theories and results, that the stroke therapeutic strategy should shift its focus from neuroprotection of neurons to protection of the neurovascular unit [1–3]. The blood-brain barrier (BBB), a vital element of the neurovascular unit, consists of brain microvessel endothelial cells (BMECs), capillary basement membranes, astrocyte endfeet, endothelia tight junctions, and pericytes [4]. Recent experimental studies indicated that BBB dysfunction was associated with many neurological diseases, such as stroke and Alzheimer's disease [4]. Cerebral ischemia and reperfusion can cause disruption to the BBB [5, 6], with damage to the components of the BBB resulting in an increase in its permeability.

Matrix metalloproteinases (MMPs), a metalloproteinase subfamily, play a crucial role in regulating the activation of growth factors, signaling molecules, and death receptors. They also attack the extracellular matrix, basal lamina, and tight junctions of endothelial cells [7]. Mounting evidence indicates that MMPs, in particular MMP-9 and -2, contribute to BBB disruption and vasogenic edema, hemorrhagic transformation, and cell death induced by cerebral ischemia and reperfusion [7–10].

Vascular endothelial growth factor (VEGF) plays an essential role in angiogenesis. However, VEGF, as a crucial vascular permeability factor, has other effects. For example, VEGF drives BBB disruption and leads to brain edema at the acute phase of stroke [11, 12], and inhibition of endogenous VEGF reduces secondary ischemic brain damage [13–15].

*Xiao-Xu-Ming* decoction (XXMD) has been widely used in China in the treatment of cerebral diseases for more than 1,000 years. Recently, XXMD has been shown to protect against cerebral ischemic injury and dementia [16–20]. However, the effects of XXMD on BBB disruption are unknown. Thus, in the present study, we sought to investigate the effects of XXMD on cerebral ischemia and reperfusion injury and to determine whether XXMD could attenuate BBB disruption by inhibiting the expression of MMP-9, MMP-2, and VEGF.

## 2. Materials and Methods

### 2.1. Preparation of XXMD

The XXMD consists of 12 crude drugs, including *Herba Ephedrae*, *cassia twig*, *Radix Paeoniae Alba*, *Rhizoma Chuanxiong*, *Radix Ginseng*, *Radix Stephaniae Tetrandrae*, *Radix Scutellariae*, *Semen Armeniacae Amarum*, *Radix Aconiti Praeparata*, *Radix Glycyrrhiza*, *Radix Ledebouriellae*, *and fresh Rhizoma Zingiberis Recens* at a ratio of 1 : 1 : 1 : 1 : 1 : 1 : 1 : 1 : 1 : 1 : 1.5 : 5. The crude drugs were purchased from TCM Pharmacy of Zhongshan Hospital, Fudan University. After the first decoction, conducted for 1 h in a 1 : 10 (w/v) drug : water ratio, the suspension was gauze filtered. Water was added for the second decoction, which lasted 1 h and which was followed by a third decoction for 1 h. The gruffs were soaked in 75% ethyl alcohol for 24 h, and the liquid was preserved. The filtered and mixed suspension from the three decoctions was collected and centrifuged at 2000 ×g for 20 min to obtain a suspension for the subsequent preparation. Dehydrated alcohol was added slowly with fast agitation until the concentration reached 75% alcohol (v/v). The solution was stirred overnight and the precipitate was discarded. The suspension and the liquid acquired from the gruffs were merged and centrifuged at 2000 ×g for 20 min, then concentrated to a final concentration of 2 g/mL (w/v). The alcohol was recovered simultaneously with a rotary evaporator. Finally, the liquid was autoclaved and stored at −20°C until use.

### 2.2. Animals

All experiments were performed on adult male Sprague-Dawley rats (Experimental Animal Center, Zhongshan Hospital, Fudan University, China) weighing 230–280 g. One hundred twenty rats were kept in groups of four and maintained on a 12:12 h light:dark cycle at a constant room temperature of 21°C with free access to food and water. The experimental protocols and animal handling procedures were approved by the Animal Care and Use Committee (ACUC) of Fudan University and were consistent with the National Institutes of Health Guide for the Care and Use of Laboratory Animals.

### 2.3. Drug Administration and Experimental Design

**Figure 1:**
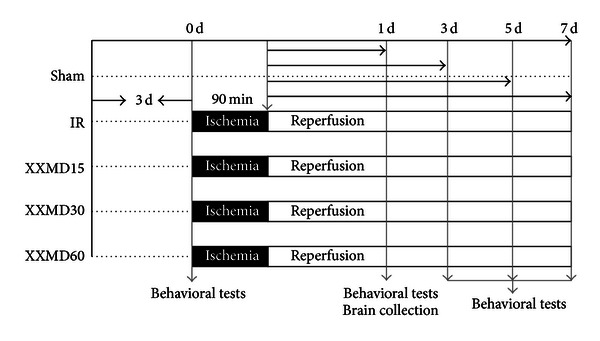
Diagram showing the experimental design of the present study. The rats (*n* = 120) were randomly divided into 5 groups. Different doses of XXMD were orally administered to rats in the XXMD-treated groups for 3 days prior to the operation until the end of the experiments. And the others were given the distilled water. The rats in the IR and XXMD-treated groups were subjected to reperfusion following 90 min of middle cerebral artery occlusion, while the rats in the sham group underwent the same surgical procedure without monofilament occlusion. Brain tissues were collected for subsequent investigations, such as TTC staining, evaluation of brain water content, BBB leakage and BBB ultrastructure changes, western blot analysis, and immunohistochemistry. In addition, behavioral tests were performed at the indicated time points.

For XXMD treatment, the common human daily dose of XXMD is 165 g/75 kg bodyweight [21]. According to the formula: *d*_rat_ = *d*_human_ × 0.7/0.11, the common dose of XXMD in rat should be 14.2 g/kg/day. In general, the drug tolerance of a rat is higher than that of human [21], we therefore selected 15, 30, and 60 g/kg/day as the low, medium, and high dosages in the present study, respectively. Rats were randomly divided into 5 groups: sham control (sham), ischemia and reperfusion (IR), and IR plus XXMD15, XXMD30, and XXMD60. The rats in the XXMD-treated groups were orally administered the corresponding doses of XXMD, and other rats were given the same volume of normal saline. All treatments were performed twice a day at 8:00 and 18:00 for 3 days before the operation and drug administration was continued until animal sacrifice at the conclusion of the experiment. The experimental design of the current study is shown in Figure 1.

### 2.4. Focal Cerebral Ischemia and Reperfusion

The focal ischemia was induced by left middle cerebral artery occlusion (MCAO) according to previously described methods with minor modifications [22, 23]. The ischemia and reperfusion induction was performed by an operator blind to the animal grouping. Briefly, the rats were anesthetized with 10% chloral hydrate (350 mg/kg, intraperitoneal (i.p.) injection). After the skin incision, the left common carotid artery (CCA) was exposed and carefully separated from nerves and tissue. The external carotid artery (ECA) and internal carotid artery (ICA) were dissected gently. The ECA was clipped, the ECA stump was stretched and aligned with the ICA, and a nylon monofilament was inserted. At this time, the microvascular clip was removed and the thread was advanced until it was about 20 mm from the CCA bifurcation. During the course of the surgery, the rectal temperature, blood gases, and cardiovascular rate of each rat were monitored and maintained. Reperfusion was initiated by withdrawal of the monofilament after 90 min of ischemia. In this study, the rats in the sham group were subjected to the same operation, but the monofilament was not inserted. The other rats in the IR group and the XXMD-treated groups underwent reperfusion following 90 min of cerebral ischemia. During the experiments, 19 rats subjected to focal cerebral ischemia and reperfusion died, and the main causes of death were cerebral infarction with hemorrhage or subarachnoid hemorrhage.

### 2.5. Quantification of Ischemic Infarct Area and Hemispheric Swelling

Twenty-four hours after reperfusion, the cerebral ischemic infarct areas were evaluated by 2,3,5-triphenyltetrazolium chloride (TTC; Sigma-Aldrich, St. Louis, MO, USA) staining. Briefly, the rats were sacrificed under deeply anesthesia, their brains were quickly removed on ice, and placed at −20°C for 15–20 min. Brains were sectioned into 6 coronal slices of 2 mm thickness and stained with 1% TTC solution in the dark for 20 min at 37°C. Finally, the tissues were fixed in 4% paraformaldehyde (in 0.1 M phosphate buffer, pH 7.4), and the percentage of cerebral infarct area was calculated 24 h later with microscope image analysis software (Image-Pro Plus, USA) according to the formula: [contralateral hemisphere area − (ipsilateral hemisphere area − infarct area)/contralateral hemisphere area] × 100%. Hemispheric swelling was assessed in slices stained by TTC according to the formula: 100%  × (ipsilateral volume − contralateral volume)/contralateral volume [24, 25].

### 2.6. Behavioral Observations

Cerebral ischemia and reperfusion can cause motor asymmetry. In the present study, behavioral observations involving three tests were performed by two investigators who were unaware of the animal grouping at different time points after reperfusion. Rats were gently handled and trained daily for 3 days before the operation to accustom the animals to being handled.

#### 2.6.1. Neurological Deficit Scores

Neurological examinations were performed at 0, 1, 3, 5, and 7 days after reperfusion. The neurological deficits were assessed on a 5-point scale described by Longa et al. [22] as follows: 0 = no deficit; 1 = failure to extend left forepaw; 2 = circling to the left; 3 = falling to the left; 4 = no spontaneous walking with a depressed level of consciousness. In the study, 5 rats that underwent MCAO without any detectable neurological deficits were excluded from the following investigations and analysis to exclude operative failures.

#### 2.6.2. Vibrissae-Elicited Forelimb Placing Test

This test was performed as previously described with slight modifications [26–28]. Briefly, when the vibrissae on each side of each rat were gently brushed against the edge of a table, they reflexively placed their forelimbs on that side onto the countertop. However, the reflex could not be induced contralateral to the ischemic injury. The reflex was tested 20 times on each side per trial, and two trials were performed per test session. If muscle tension or struggling occurred, the rats were held and stroked until they relaxed and the trial was resumed. The number of vibrissae stimulations in which a paw placement occurred was counted, and the percentage was calculated.

#### 2.6.3. Tail Hang Test

Tail hang tests were assessed at different time points according to the method described by Zhao et al. and Borlongan et al. [27, 29] with minor modifications. Each rat was lifted 5–10 cm above the table. An ischemia-damaged rat will immediately turn to the right side. The large right turn is accompanied by a twisting of the body and a raise of the head toward the holding hand. “Turns” were counted when the angle reached 90°C or more. Smaller turns were not counted. The rat was lifted no more than 5 s on each trial and was released for a few seconds before the next trial. The test was repeated 20 times per testing day. The percentage of trials during which a right turn occurred was calculated.

### 2.7. Brain Water Content Measurement

Brain water content was measured with the dry-wet weight method 24 h after reperfusion. After being anesthetized, the animals were sacrificed, and the brain tissues were removed and separated into ischemic and nonischemic hemispheres, which were immediately weighed to obtain the wet weight (WW). Then the tissues were placed in an oven at 100°C for 24 h and re-weighed to obtain the dry weight (DW). The brain water content was assessed with the following formula: 100%  × (WW−DW)/WW.

### 2.8. BBB Integrity

To measure BBB permeability, the Evans blue content was assessed. Briefly, 2% Evans blue solution (4 mL/kg), dissolved in normal saline, was administered intravenously at 23 h after reperfusion. The rats were deeply anesthetized 1 h later and transcardially perfused with normal saline to wash away the remaining dye in the blood vessels. Ischemic and nonischemic hemispheres were dissected and weighed. The ischemic brain tissues were placed in formamide (1 mL/100 mg) at 55°C for 24 h. Finally, the absorption of the tissue was calculated at the 620 nm wavelength with a luminescence spectrometer (Flex Station 3, Molecular Devices, Sunnyvale, CA, USA) according to the standard curve of Evans blue content, with formamide as negative control.

### 2.9. Ultrastructural Alterations of BBB

Transmission electron microscopy was used to determine BBB ultrastructural alterations. Rats were deeply anesthetized and transcardially perfused by normal saline followed by cold 1% glutaraldehyde/3% paraformaldehyde solution 24 h after reperfusion. The brain tissues were removed and postfixed in the above solution. Coronal sections of 1.0 m^3^ in volume and located 1.2 mm to 0.2 mm rostral to bregma were prepared to obtain the peri-infarct tissue of the ipsilateral cortex. The tissue was postfixed in 2% osmium for 1.5 h, dehydrated, and embedded in Epon 812 Resin (TAAB, Berks, UK). Ultrathin (0.06 *μ*m) sections were cut with a diamond knife, stained with uranyl acetate and lead citrate, and observed with an electron microscope (H-7100S; Hitachi, Tokyo, Japan).

### 2.10. Western Blotting Analysis

Western blotting was used to assess the expression levels of MMP-9, MMP-2, and VEGF 24 h after cerebral ischemia and reperfusion. The ischemic hemisphere tissues were prepared in lysis buffer with protease inhibitors (Beyotime, Haimen, Jiangsu, China) and centrifuged at 13,000 ×g for 5 min. The supernatant was collected. Protein concentrations were determined with a BCA kit (Beyotime). Fifty micrograms of protein solution were separated by electrophoresis in different concentrations of polyacrylamide gel and transferred to polyvinylidene fluoride membranes (Millipore, Bedford, MA, USA). After blocking for 2 h with a 5% solution of skim milk, prepared with Tris-buffered saline with 0.1% Tween-20 (TBST), the primary antibodies polyclonal rabbit anti-MMP-9, -MMP-2, and -VEGF (all diluted 1 : 1000; Abcam, HK, China) were incubated with the membranes at 4°C overnight. The membranes were incubated with the secondary antibody conjugated with horseradish-peroxidase (Beyotime) after washing with TBST three times for 10 min each. The targeted antigens were detected by standard chemical luminescence methods (Beyotime) with Fluor Chem FC2 gel imaging system (Alpha Innotech, Santa Clara, CA, USA). The expression of the targeted proteins was determined by using the GADPH protein as a loading control. Western blots were duplicated with three independent sets. Band intensities were measured with Quantity One software (Bio-Rad Laboratories, Hercules, CA, USA).

### 2.11. Immunohistochemistry

Immunohistochemical staining was used to evaluate whether treatment with XXMD changes the expression of MMP-9, MMP-2, and VEGF after cerebral ischemia and reperfusion. Rat brain tissues were postfixed in 4% paraformaldehyde for 24 h and then immersed in 30% sucrose solution with phosphate buffer saline (PBS, pH 7.4) for 24 h. Coronal sections (10 *μ*m thick) at the level of the anterior commissure in the infarct region were obtained. For immunohistochemistry, sections were deparaffinized and incubated with 0.3% H_2_O_2_ in PBS. After blocking with 5% normal goat serum, the sections were incubated with rabbit polyclonal anti-MMP-9 antibody (diluted 1 : 100; Abcam), anti-MMP-2 antibody (diluted 1 : 300; Abcam), or anti-VEGF antibody (diluted 1 : 100; Abcam) at 4°C overnight. After washing in PBS, the sections were incubated with the secondary antibody conjugated with horseradish-peroxidase (Beyotime) for 1 h at 37°C, and then visualized using 3,3′-diaminobenzidine tetrahydrochloride (DAB kit; Beyotime) The sections were photographed and observed with a light microscope (Olympus/BX51, Tokyo, Japan).

### 2.12. Statistical Analysis

Values are shown as the means ± standard error of the mean (SEM). Differences in quantitative data obtained from cerebral infarct area, hemispheric swelling, behavioral tests, brain water content, BBB integrity, and integrated densities of protein bands from western blots were evaluated by one-way analysis of variance (ANOVA) followed by Student's *t*-test and post hoc Fisher's tests. *P* < 0.05 was considered statistically significant. Statistical analysis was performed with SPSS version 11.5 for Windows (SPSS, Chicago, IL, USA).

## 3. Results

### 3.1. XXMD Reduced Cerebral Infarct Area and Hemispheric Swelling

**Figure 2:**
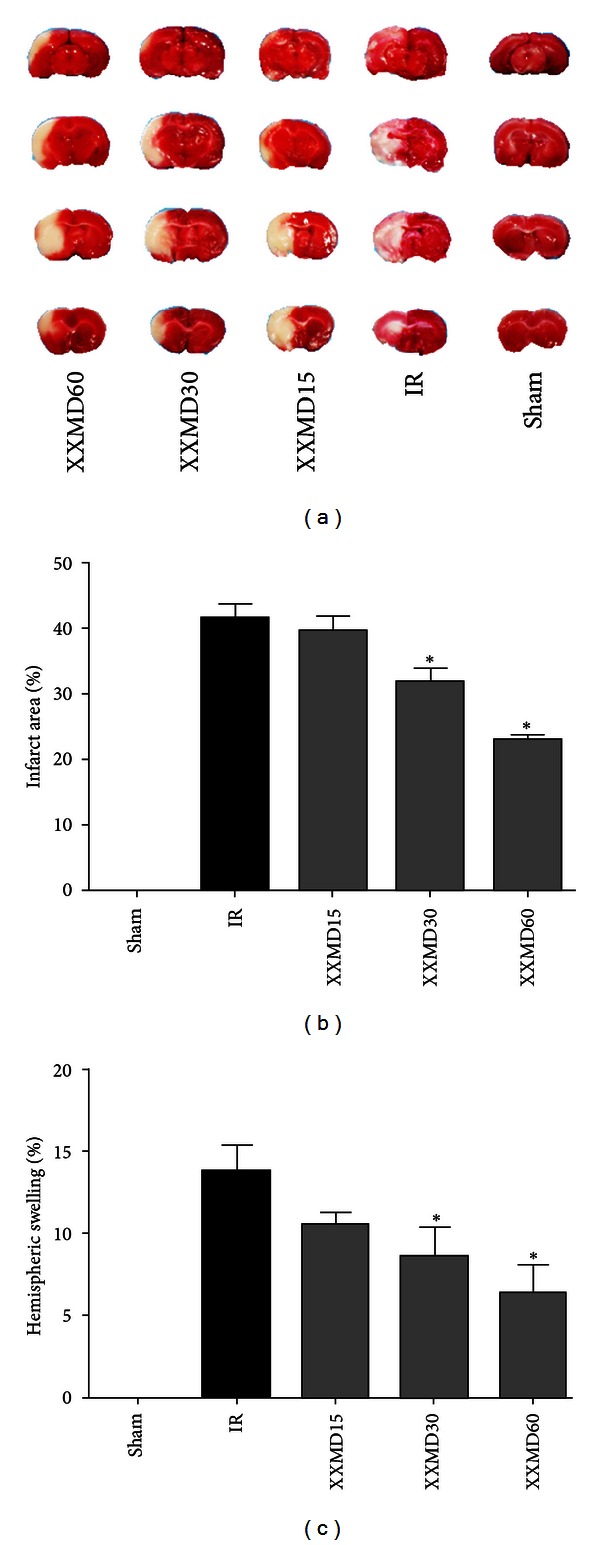
Effects of XXMD on cerebral infarct area and hemispheric swelling 24 h after reperfusion. (a) Representative images of TTC-stained brain slices. (b) The quantitative analysis of cerebral infarct area. (c) The quantitative analysis of hemispheric swelling. The images indicated that XXMD remarkably reduced cerebral infarct area and hemispheric swelling. Data are reported as the mean ± SEM, *n* = 4 for each group.*P < 0.05 versus the IR group.

Cerebral infarct area and hemispheric swelling, induced by MCAO, were evaluated through the use of TTC staining. XXMD (30 g/kg/day and 60 g/kg/day) significantly reduced both the infarct area in the territory of the middle cerebral artery and hemispheric swelling compared to the IR group (Figure 2).

### 3.2. XXMD Improved Neurological Function

**Figure 3:**
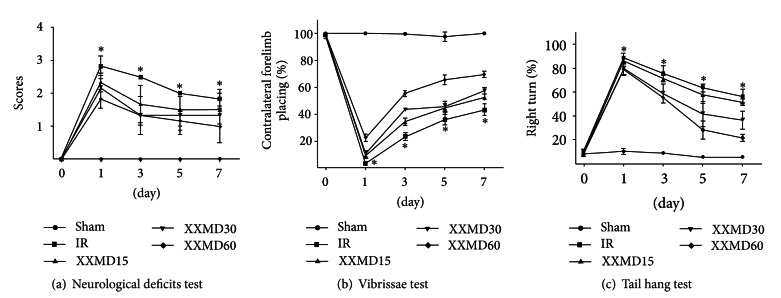
The behavioral tests of the 5 groups at different time points. (a) The neurological deficits test. (b) The vibrissae test. (c) The tail hang test. The results indicated that XXMD significantly improved neurological function in injured rats. Data represent the mean ± SEM, *n* = 4-5 for each group. *P < 0.05 versus the XXMD30 and XXMD60 groups.

The effects of XXMD on behavioral tests after reperfusion were studied. Compared to the IR group, the XXMD30 and XXMD60 treatment had significantly reduced neurological deficit scores at 0, 1, 3, 5, and 7 days after reperfusion (Figure 3(a)). The forelimb placing of the nonischemic hemisphere induced by vibrissae brushing was disrupted and gradually improved from 3 days to 7 days after stroke induction. However, XXMD30 and XXMD60 treatment notably decreased the number of contralateral forelimb placements from 1 to 7 days after reperfusion (Figure 3(b)). In the tail test, the percentage of large right turns was significantly attenuated in the XXMD30 and XXMD60 groups compared to the IR group (Figure 3(c)). Thus, these results indicated that XXMD improved neurological function and motor asymmetry induced by ischemia and reperfusion. 

### 3.3. XXMD Reduced Brain Water Content and BBB Disruption

**Figure 4:**
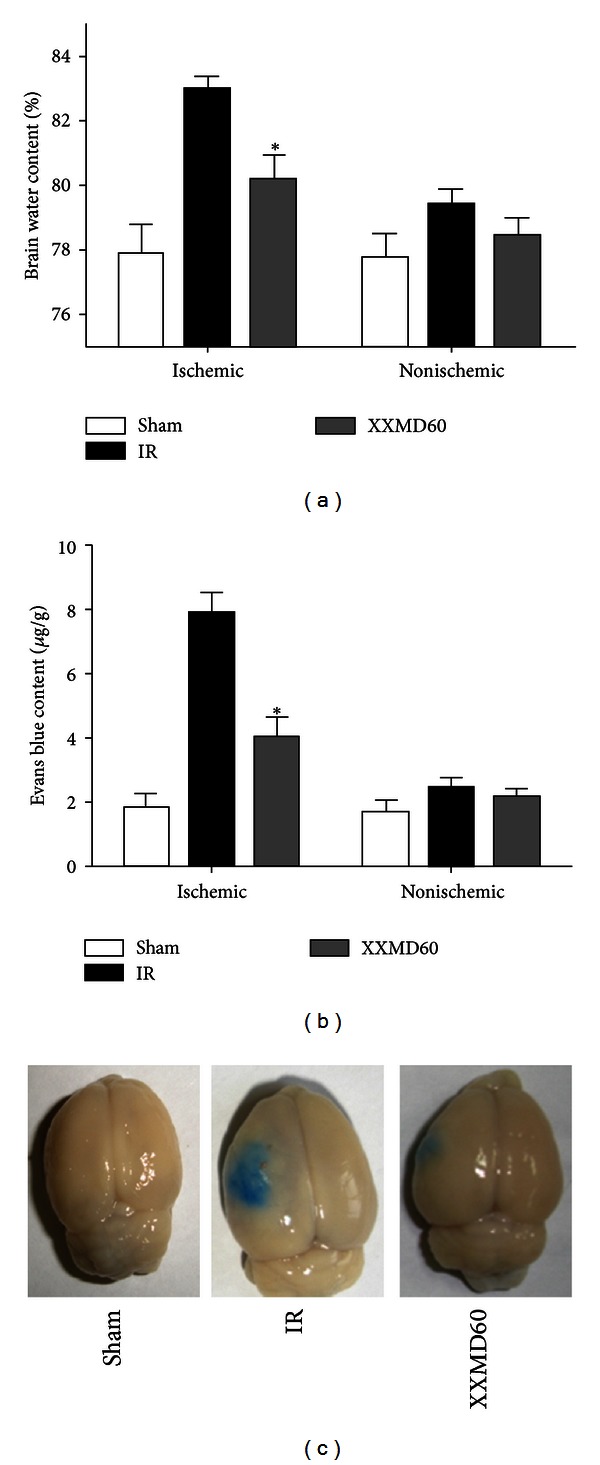
Brain water content and Evans blue content 24 h after reperfuison. (a) The quantitative analysis of brain water content. (b) The quantitative analysis of Evans blue content. (c) Representative images of Evans blue extravasation. The results indicated that XXMD (60 g/kg/day) treatment attenuated the increase in brain water content and Evans blue content induced by cerebral ischemia and reperfusion. Data are reported as the mean ± SEM, *n* = 4 for each group. *P < 0.05 versus the IR group.

Brain water content and Evans blue content were evaluated 24 h after reperfusion. The brains of rats administered XXMD (60 g/kg/day) had a lower water content than those of the IR group. The nonischemic hemispheres were not significantly different between groups. The Evans blue content results were similar to those of the brain water content. BBB disruption occurred and was remarkably increased 24 h after reperfusion. XXMD treatment (60 g/kg/day) significantly decreased Evans blue content compared with the IR group (3.99 ± 0.67 *μ*g/g tissue versus 7.89 ± 0.68 *μ*g/g tissue) in the ischemic hemisphere. However, there were no significant differences between the nonischemic hemispheres of different groups (Figure 4).

### 3.4. XXMD Attenuated the Changes in the BBB Ultrastructure

**Figure 5:**
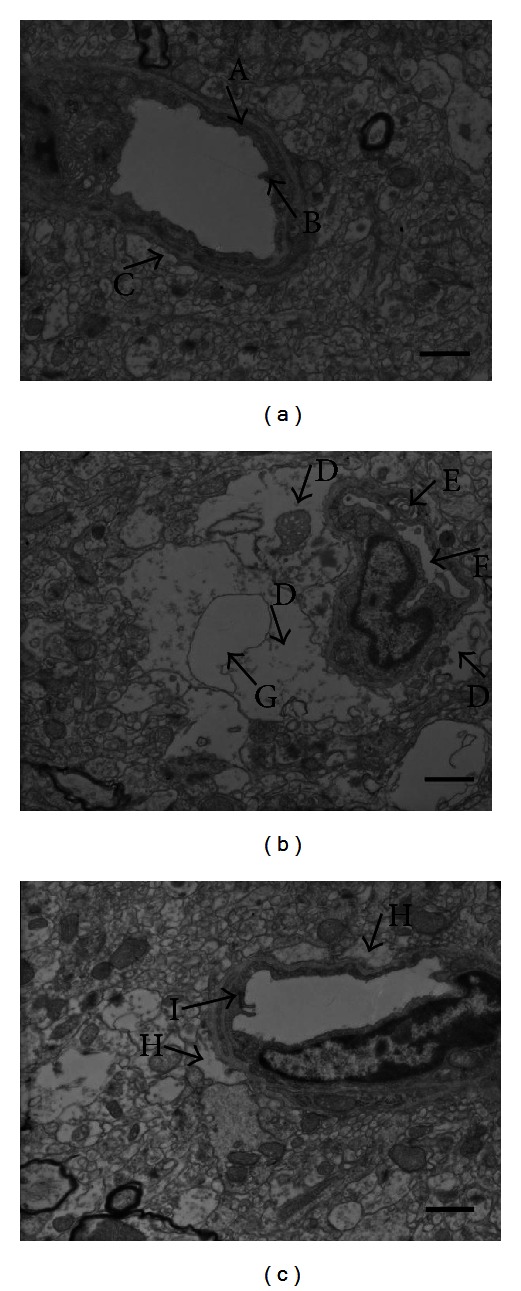
The ultrastructural variations of the BBB 24 h after reperfusion. (a) The ultrastructure of the BBB in the sham group. Strong integrity of the basement membrane (A), a clear tight junction (B), and normal astrocyte endfeet (C) were observed. (b) The ultrastructure of the BBB in the IR group. The edema around the capillary was marked in the IR group, the astrocyte endfeet surrounding the capillaries were swollen (D), and some endfeet showed vacuolar changes. Moreover, the Golgi complex was swollen (E), the capillary lumen was shrunk (F), and the tight junction could not be observed in the images. (c) The ultrastructure of the BBB in the XXMD60 group. The edema was mitigated (H) and a regular capillary lumen, continuous base membrane, and clear tight junction (I) could be found. *n* = 4 for each group. Scale bar = 1 *μ*m.

Transmission electron microscopy was used to detect BBB ultrastructural alterations. The cortical microvessels in the sham group were normal, with a continuous basal lamina, regular endothelial cells, and astrocyte endfeet. However, a series of changes indicative of BBB disruption were observed in the IR group. For example, the capillary lumen was deflated and edema was easily detected around the capillary. In addition, astrocyte endfeet surrounding the capillaries appeared markedly swollen, and some showed vacuolar changes. The tight junctions between endothelial cells were unclear, and swollen Golgi complexes were observed. These damages were alleviated in the XXMD60 group; the integrity of the capillary endothelium was almost normal with a regular capillary lumen and continuous base membrane. Furthermore, the edema around the capillary and the swelling of astrocyte foot processes were decreased (Figure 5).

### 3.5. XXMD Downregulated the Expression of MMP-9, MMP-2, and VEGF

**Figure 6:**
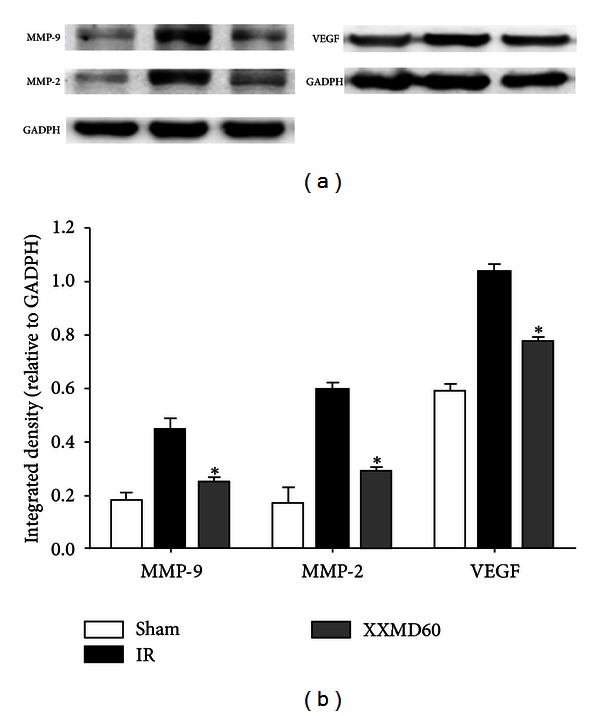
Effects of XXMD on MMP-9, MMP-2, and VEGF expressions 24 h after reperfusion. (a) Representative protein bands from western blotting for MMP-9, -2, and VEGF in the penumbra of the ischemic cortex. (b) Integrated density for MMP-9,-2, and VEGF. The expression levels of MMP-9, MMP-2, and VEGF under XXMD treatment were significantly reduced compared to the IR group. Data are reported as the mean ± SEM, *n* = 3 for each group. *P < 0.05 versus the IR group.

Western blotting was used to investigate the expression levels of MMP-9, MMP-2, and VEGF 24 h after reperfusion. The results and subsequent analysis of the western blots showed that MMP-9, MMP-2, and VEGF were increased after ischemia and reperfusion. However, XXMD treatment (30 and 60 g/kg/day) blocked the increases in MMP-9, MMP-2, and VEGF expressions (Figure 6), suggesting that reducing the increased levels of these proteins may be one of the mechanisms underlying the neuroprotection of XXMD against BBB disruption and ischemic injury.

### 3.6. XXMD Reduced the Distribution of MMP-9, MMP-2, and VEGF

**Figure 7:**
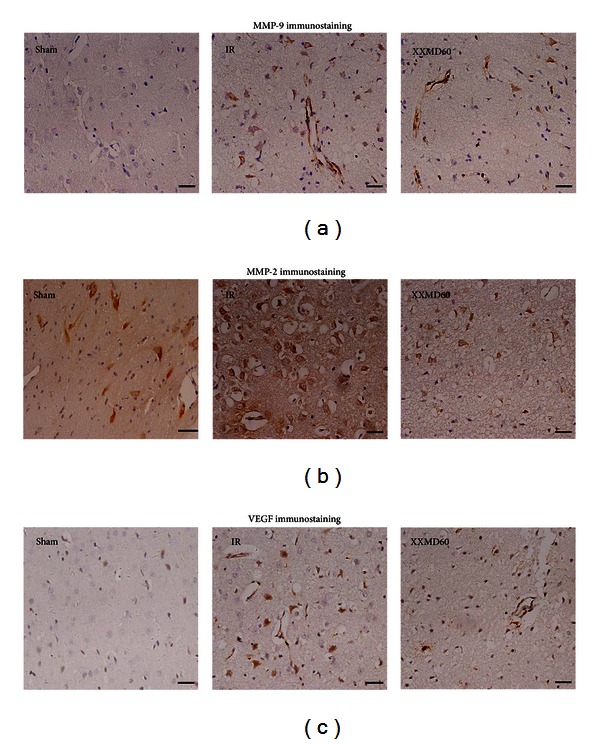
MMP-9, MMP-2, and VEGF immunohistochemistry in the ischemic cortex 24 h after reperfusion. (a) Representative images of MMP-9 immunohistochemistry in different groups. The images showed intense staining of neurons, glial cells, and cerebral vessels after ischemia and reperfusion. However, there were fewer stained neurons and glial cells in the XXMD60 group than in the IR group. (b) Representative images of MMP-2 immunohistochemistry. In the sham group, there was less staining in the cortex. MMP-2 immunostaining was enhanced 24 h after reperfusion in the IR group. The intensity of the MMP-2 staining was reduced in the XXMD60 group, with only a small number of strongly stained neurons and astrocytes. (c) Representative images of VEGF immunohistochemistry. There was almost no staining in the cortex in the sham group. VEGF immunostaining was intensely increased in ischemic cerebral vessels, neurons and reactive astrocytes in the IR group. In the XXMD treatment groups, VEGF immunostaining was reduced in intensity with a similar pattern for cell bodies and processes as in the IR group. *n* = 6, scale bar = 20 *μ*m.

The next experiments were used to study the distribution of MMP-9, MMP-2, and VEGF in the ischemic cortex by immunohistochemistry. Extensive ischemic damage was evident in these regions. In the sham group, there was almost no MMP-9 immunostaining in the cortex. However, in the IR group, it was intense and mainly localized to the cytoplasm of neurons and glial cells, though cerebral vessels were also notably stained. Most cells showed ischemic changes. However, MMP-9 staining was less intense in the XXMD60 group compared with the IR group (Figure 7(a)).

The MMP-2 immunostaining results were similar to those of MMP-9. In the sham groups, MMP-2 immunostaining was mainly localized to the cytoplasm of a few glial cells and to neurons with normal morphological appearance. Twenty-four hours after reperfusion, MMP-2 immunostaining was increased and many neurons in the ischemic cortex showed ischemic changes, such as shrinkage of the nucleus and cytoplasm, in an area in which many ischemic neurons, glial cells, and cerebral vessels were clearly stained. Although MMP-2 staining was intense in the XXMD60 group, the extent was reduced compared to that of the IR group, and the majority of positive cells were glial cells and neurons, the number of which was less than the IR group (Figure 7(b)).

VEGF immunostaining was absent in the sham rat cortex. However, in the IR group, VEGF immunostaining was increased and intensely located in ischemic cerebral vessels, neurons, and reactive astrocytes. VEGF immunostaining was weaker in the XXMD60 group than in the IR group (Figure 7(c)).

## 4. Discussion

In the present study, we demonstrated that XXMD treatment significantly attenuated neurological injury induced by cerebral ischemia and reperfusion. XXMD reduced the cerebral infarct area and hemispheric swelling and improved behavioral function. Furthermore, the data showed that XXMD treatment attenuated BBB disruption, which may be associated with the downregulation of the expression of MMP-9, MMP-2, and VEGF. XXMD may protect against cerebral ischemia and reperfusion injury by reducing the levels of these proteins.

BBB, which is essential for the function of the central nervous system, plays an important role in the maintenance of homeostasis [30]. BBB disruption is induced at the onset of cerebral ischemia and reperfusion. When BBB breakdown occurs, many serum proteins that are detrimental to neurons pass through the barrier, further worsening brain injury. The data of the present study consistently indicated that Evans blue content, as an indicator of BBB permeability, distinctly increased and that brain edema rapidly formed at the early stage of ischemia and reperfusion injury. These events were significantly blocked by XXMD treatment. The crucial components of the BBB, such as astrocytes and basement membranes, and the primary barrier, which is formed by endothelia cells in capillaries, contribute to the integrity and physical function of the BBB [31]. However, a series of changes rapidly occur after reperfusion. The transmission electron microscope images showed marked edema of astrocyte endfeet and crushed microvessels, changes that eventually lead to microcirculation dysfunction. XXMD treatment substantially alleviated the morphological changes and blocked the decrease in brain blood flow. Therefore, we conclude that XXMD protected against disruption to both the structure and function of the BBB.

Following reperfusion, MMPs and free radicals that may mediate the attack on the capillaries are released [10, 31]. The increase in MMP-9 and -2 degrades tight junction proteins that strengthen the endothelial cell wall and form the endothelial barrier. Degradation of these proteins results in BBB disruption, further vasogenic edema formation, and neuronal damage in stroke with reperfusion [10, 32, 33].

Angiogenesis is a potent process in stroke. VEGF, a substantial mediator of angiogenesis, plays bidirectional roles in different stages of cerebral ischemia and reperfusion. It markedly enhances angiogenesis and reduces neurological deficits during the recovery stage [34, 35]. However, it not only increases BBB permeability and the incidence of hemorrhagic transformation but also aggravates secondary ischemic insults at the acute stage of stroke [36, 37]. Like MMP-9 and -2, VEGF increases the permeability of the BBB due to tight junction disassembly [38].

**Figure 8:**
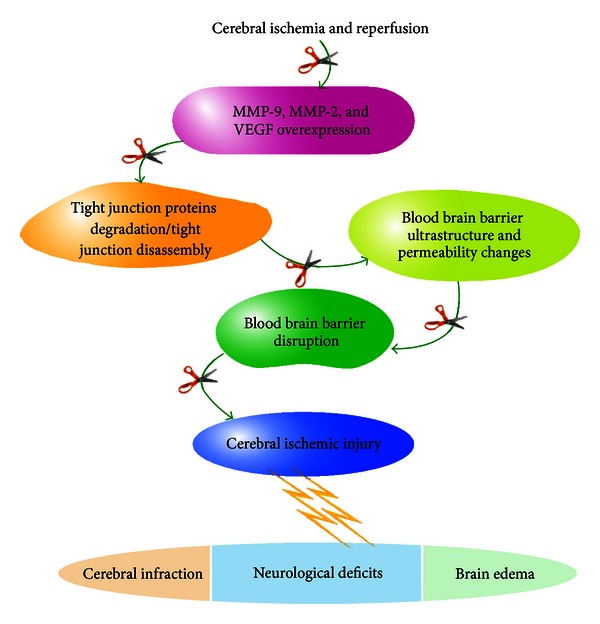
The mechanisms underlying the neuroprotection of XXMD against BBB disruption and ischemic injury induced by focal cerebral ischemia and reperfusion. Briefly, focal cerebral ischemia and reperfusion resulted in increase in MMP-9, MMP-2, and VEGF at the early stage of cerebral ischemia and reperfusion, which degraded the tight junction proteins or led to tight junction disassembly, further caused changes to the BBB ultrastructure and increased BBB permeability. Eventually, these alterations led to BBB disruption and ischemic injury, which includes cerebral infarction, neurological deficits, and brain edema. Interestingly, XXMD administration notably downregulated the expression levels of MMP-9, -2, and VEGF, further enhanced the tight junction and minimized BBB ultrastructure and permeability changes. As a result, XXMD administration inhibited BBB disruption and alleviated ischemic injury. The open scissors indicate the effects of XXMD treatment on cerebral injury following stroke and reperfusion.

Mounting evidence indicates that VEGF upregulates the releases and expressions of MMP-9 and -2 [39–42]. In the current study, we studied the expression levels and distribution of MMP-9, MMP-2, and VEGF at the same time point as for Evans blue leakage. The data showed that MMP-9, MMP-2, and VEGF proteins were upregulated and markedly expressed by neurons, astrocytes, and cerebral vessels 24 h after reperfusion. XXMD treatment significantly inhibited the expressions of MMP-9, MMP-2, and VEGF. These results support the view that MMP-9, MMP-2, and VEGF are involved in the BBB breakdown after cerebral ischemia and reperfusion. Thus, the data further suggested that XXMD preserved BBB integrity after reperfusion which may be partly through blocking MMP-9, MMP-2 and VEGF expressions (Figure 8). 

In conclusion, the current study demonstrated that XXMD, as a drug of multiple targets, improved neurological function and exerted a protective effect on the BBB by downregulation of MMP-9, -2, and VEGF after cerebral ischemia and reperfusion injury.

## References

[1] Del GZ (2006). Stroke and neurovascular protection. *The New England Journal of Medicine*.

[2] Lo EH, Dalkara T, Moskowitz MA (2003). Mechanisms, challenges and opportunities in stroke. *Nature Reviews Neuroscience*.

[3] Lok J, Gupta P, Guo S (2007). Cell-cell signaling in the neurovascular unit. *Neurochemical Research*.

[4] Ballabh P, Braun A, Nedergaard M (2004). The blood-brain barrier: an overview: structure, regulation, and clinical implications. *Neurobiology of Disease*.

[5] Kuroiwa T, Ting P, Martinez H, Klatzo I (1985). The biphasic opening of the blood-brain barrier to proteins following temporary middle cerebral artery occlusion. *Acta Neuropathologica*.

[6] Belayev L, Busto R, Zhao W, Ginsberg MD (1996). Quantitative evaluation of blood-brain barrier permeability following middle cerebral artery occlusion in rats. *Brain Research*.

[7] Rosenberg GA (2009). Matrix metalloproteinases and their multiple roles in neurodegenerative diseases. *The Lancet Neurology*.

[8] Lucivero V, Prontera M, Mezzapesa DM (2007). Different roles of matrix metalloproteinases-2 and -9 after human ischaemic stroke. *Neurological Sciences*.

[9] Montaner J, Alvarez-Sabín J, Molina CA (2001). Matrix metalloproteinase expression is related to hemorrhagic transformation after cardioembolic stroke. *Stroke*.

[10] Rosenberg GA, Estrada EY, Dencoff JE (1998). Matrix metalloproteinases and TIMPs are associated with blood-brain barrier opening after reperfusion in rat brain. *Stroke*.

[11] Argaw AT, Asp L, Zhang J (2012). Astrocyte-derived VEGF-A drives blood-brain barrier disruption in CNS inflammatory disease. *Journal of Clinical Investigation*.

[12] Zhang ZG, Zhang L, Jiang Q (2000). VEGF enhances angiogenesis and promotes blood-brain barrier leakage in the ischemic brain. *Journal of Clinical Investigation*.

[13] Chiba Y, Miyake TSS, Koyama J, Kondoh T, Hosoda K, Kohmura E (2008). Anti-VEGF receptor antagonist (VGA1155) reduces infarction in rat permanent focal brain ischemia. *Kobe Journal of Medical Sciences*.

[14] Chi OZ, Hunter C, Liu X, Weiss HR (2007). Effects of anti-VEGF antibody on blood-brain barrier disruption in focal cerebral ischemia. *Experimental Neurology*.

[15] Koyama J, Miyake S, Sasayama T, Chiba Y, Kondoh T, Kohmura E (2010). The novel VEGF receptor antagonist, VGA1155, reduces edema, decreases infarct and improves neurological function after stroke in rats. *Kobe Journal of Medical Sciences*.

[16] Du K, Wu C, Ding C, Zhao S, Qin H, Zhang J (2009). Simultaneous LC-MS analysis and of wogonin and oroxylin a in rat plasma, and pharmacokinetic studies after administration of the active fraction from *Xiao-Xu-Ming* decoction. *Chromatographia*.

[17] Wang Y, Ding C, Du K (2009). Identification of active compounds and their metabolites by high-performance liquid chromatography/electrospray ionization Fourier transform ion cyclotron resonance mass spectrometry from *Xiao-Xu-Ming* decoction (XXMD). *Rapid Communications in Mass Spectrometry*.

[18] Li Z, Ni K, Du G (2007). Simultaneous analysis of six effective components in the anti-Alzheimer’s disease effective component group of *Xiao-Xu-Ming* decoction. *Chinese Journal of Chromatography*.

[19] Cai DF, Yang YK, Gu XX (2007). Clinical trial on treatment of acute cerebral infarction with TCM treatment according to syndrome differentiation combining Western medicine by staging. *Chinese Journal of Integrated Traditional and Western Medicine*.

[20] Shi XM, Cai DF, Dai W (2003). Effect of QFTL TCP on endothelin and gene expression in acute cerebral ischemia. *China Jounal of Modern Medicine*.

[21] Zhu XH, Li SJ, Hu HH, Sun LR, Das M, Gao TM (2010). Neuroprotective effects of *Xiao-Xu-Ming* decoction against ischemic neuronal injury in vivo and in vitro. *Journal of Ethnopharmacology*.

[22] Longa EZ, Weinstein PR, Carlson S, Cummins R (1989). Reversible middle cerebral artery occlusion without craniectomy in rats. *Stroke*.

[23] Bigdeli MR, Hajizadeh S, Froozandeh M, Rasulian B, Heidarianpour A, Khoshbaten A (2007). Prolonged and intermittent normobaric hyperoxia induce different degrees of ischemic tolerance in rat brain tissue. *Brain Research*.

[24] Huang Z, Huang PL, Panahian N, Dalkara T, Fishman MC, Moskowitz MA (1994). Effects of cerebral ischemia in mice deficient in neuronal nitric oxide synthase. *Science*.

[25] Li S-Y, Yang D, Fu ZJ, Woo T, Wong D, Lo ACY (2012). Lutein enhances survival and reduces neuronal damage in a mouse model of ischemic stroke. *Neurobiology of Disease*.

[26] Schallert T, Fleming SM, Leasure JL, Tillerson JL, Bland ST (2000). CNS plasticity and assessment of forelimb sensorimotor outcome in unilateral rat models of stroke, cortical ablation, parkinsonism and spinal cord injury. *Neuropharmacology*.

[27] Zhao H, Shimohata T, Wang JQ (2005). Akt contributes to neuroprotection by hypothermia against cerebral ischemia in rats. *Journal of Neuroscience*.

[28] Gao X, Zhang H, Takahashi T (2008). The Akt signaling pathway contributes to postconditioning’s protection against stroke; the protection is associated with the MAPK and PKC pathways. *Journal of Neurochemistry*.

[29] Borlongan CV, Tajima Y, Trojanowski JQ, Lee VMY, Sanberg PR (1998). Transplantation of cryopreserved human embryonal carcinoma-derived neurons (NT2N cells) promotes functional recovery in ischemic rats. *Experimental Neurology*.

[30] Hawkins BT, Davis TP (2005). The blood-brain barrier/neurovascular unit in health and disease. *Pharmacological Reviews*.

[31] Ji HH, Sang WH, Lee SK (2005). Free radicals as triggers of brain edema formation after stroke. *Free Radical Biology and Medicine*.

[32] Liu J, Jin X, Liu KJ, Liu W (2012). Matrix metalloproteinase-2-mediated occludin degradation and caveolin-1-mediated claudin-5 redistribution contribute to blood-brain barrier damage in early ischemic stroke stage. *Journal of Neuroscience*.

[33] Yang Y, Rosenberg GA (2011). MMP-mediated disruption of claudin-5 in the blood-brain barrier of rat brain after cerebral ischemia. *Methods in Molecular Biology*.

[34] Yang JP, Liu HJ, Liu XF (2010). VEGF promotes angiogenesis and functional recovery in stroke rats. *Journal of Investigative Surgery*.

[35] Wang YQ, Cui HR, Yang SZ (2009). VEGF enhance cortical newborn neurons and their neurite development in adult rat brain after cerebral ischemia. *Neurochemistry International*.

[36] Li Z, Wang R, Li S (2008). Intraventricular pre-treatment with rAAV-VEGF induces intracranial hypertension and aggravates ischemic injury at the early stage of transient focal cerebral ischemia in rats. *Neurological Research*.

[37] Chi OZ, Hunter C, Liu X, Weiss HR (2008). Effects of deferoxamine on blood-brain barrier disruption and VEGF in focal cerebral ischemia. *Neurological Research*.

[38] Wang W, Dentler WL, Borchardt RT (2001). VEGF increases BMEC monolayer permeability by affecting occludin expression and tight junction assembly. *The American Journal of Physiology*.

[39] Wang H, Keiser JA (1998). Vascular endothelial growth factor upregulates the expression of matrix metalloproteinases in vascular smooth muscle cells: role of flt-1. *Circulation Research*.

[40] Lamoreaux WJ, Fitzgerald MEC, Reiner A, Hasty KA, Charles ST (1998). Vascular endothelial growth factor increases release of gelatinase A and decreases release of tissue inhibitor of metalloproteinases by microvascular endothelial cells in vitro. *Microvascular Research*.

[41] Rooprai HK, Rucklidge GJ, Panou C, Pilkington GJ (2000). The effects of exogenous growth factors on matrix metalloproteinase secretion by human brain tumour cells. *British Journal of Cancer*.

[42] Valable S, Montaner J, Bellail A (2005). VEGF-induced BBB permeability is associated with an MMP-9 activity increase in cerebral ischemia: both effects decreased by Ang-1. *Journal of Cerebral Blood Flow and Metabolism*.

